# Modeling for IFOG Vibration Error Based on the Strain Distribution of Quadrupolar Fiber Coil

**DOI:** 10.3390/s16071131

**Published:** 2016-07-21

**Authors:** Zhongxing Gao, Yonggang Zhang, Yunhao Zhang

**Affiliations:** College of Automation, Harbin Engineering University, Harbin 150001, China; zhongxing1141@sina.com (Z.G.); zhangyunhao@hrbeu.edu.cn (Y.Z.)

**Keywords:** fiber elastic modulus, quadrupolar fiber coil, vibration model, asymmetry of strain distribution

## Abstract

Improving the performance of interferometric fiber optic gyroscope (IFOG) in harsh environment, especially in vibrational environment, is necessary for its practical applications. This paper presents a mathematical model for IFOG to theoretically compute the short-term rate errors caused by mechanical vibration. The computational procedures are mainly based on the strain distribution of quadrupolar fiber coil measured by stress analyzer. The definition of asymmetry of strain distribution (ASD) is given in the paper to evaluate the winding quality of the coil. The established model reveals that the high ASD and the variable fiber elastic modulus in large strain situation are two dominant reasons that give rise to nonreciprocity phase shift in IFOG under vibration. Furthermore, theoretical analysis and computational results indicate that vibration errors of both open-loop and closed-loop IFOG increase with the raise of vibrational amplitude, vibrational frequency and ASD. Finally, an estimation of vibration-induced IFOG errors in aircraft is done according to the proposed model. Our work is meaningful in designing IFOG coils to achieve a better anti-vibration performance.

## 1. Introduction

Interferometric fiber optic gyroscope (IFOG) is an optical sensor based on Sagnac effect, which is used to determine the angular velocity of the carrier [1]. Over the past few decades, it has been developed as a leading instrument in sensing of rotational motion and has been manufactured around the world for a wide range of both military and commercial applications [2,3]. Some commercial IFOG with high performance has been reported in recent years [4,5], the bias stability specification over environment is 0.0005°/h with the scale factor less than 10 ppm and an angle random walk (ARW) < 8 × 10^−5.0^/h^1/2^. A key component in IFOG is fiber coil whose winding quality will affect the performance of gyroscope directly. Thus far, numerous researches have been performed to improve the adaptability of IFOG in the harsh environment. The theoretical model for thermal induced rate error of IFOG with temperature ranging from −40 °C to 60 °C is established in [6], which can be potentially used in the temperature compensation for gyroscope. To improve the thermal performance of the fiber-optic gyroscope, the approach of using air-core photonic-bandgap fiber is present in [7] and it is demonstrated by experiments in [8] that the thermal sensitivity is 6.5 times less than the conventional fiber coil. Furthermore, modeling for rate errors of IFOG induced by moisture diffusion is present in [9]. In practical applications for high precision IFOG, mechanical vibration is a common and inevitable environmental factor that typically encountered in the inertial navigation platforms, and it will degrade the performance of the gyroscope seriously. Generally, the anti-vibration performance of IFOG can be effectively improved by optimizing the mechanical structure design, avoiding the resonant frequency, and tightening the optical components specifically [10,11,12]. Nevertheless, these preliminary methods cannot reveal the principles of vibration error in essence. In other words, a systematic study to analyze the vibration error of IFOG has not been thoroughly addressed and that is the main purpose of our work.

The vibrational problem of IFOG is rather complicated and tough in practical engineering. In other words, it is very difficult to establish a mathematical model precisely to describe and analyze the vibration error of gyroscope due to various disturbances such as imperfect fiber coil and inaccurate parameters for computation [13]. Furthermore, previous work in [14] mainly focus on the vibration error from the perspective of control equation of IFOG as well as signal processing in circuit, but not considered the error induced by imperfect winding of fiber coil. To the best of our knowledge, this is the first report to investigate IFOG vibration error in terms of strain distribution in the quadrupolar (QAD) fiber coil. The paper is organized as follows: The theory of closed-loop IFOG is introduced in Section 2. The mathematical model of IFOG rate error induced by vibration is established in Section 3. The computational results based on the model are provided in Section 4. Finally, the conclusion and discussion are given in the last section.

## 2. Description of Closed-Loop IFOG

**Figure 1:**
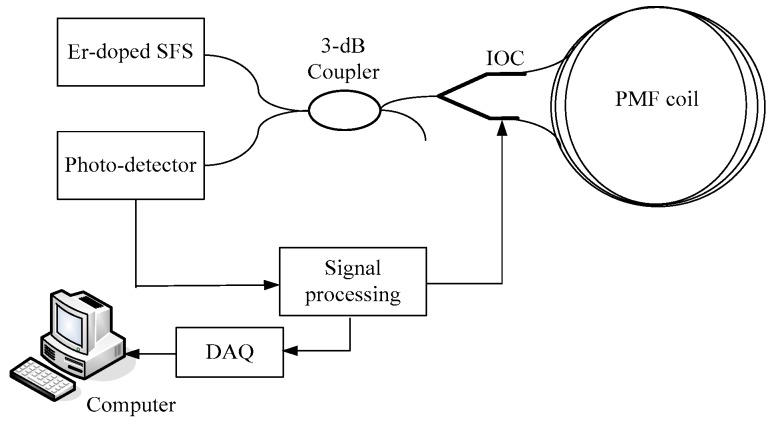
Schematic diagram of digital closed-loop interferometric fiber optic gyroscope (IFOG).

The details of the optical components and the signal processing circuit constructed for closed-loop IFOG are introduced in Figure 1. To achieve higher output power and better wavelength stability, a broadband Er-doped super fluorescent fiber source (SFS) is utilized as the light source. The 3-dB coupler splits the input light into two waves to propagate in the fiber coil. The Sagnac interferometer consists of an integrated-optic circuit (IOC) and a polarization maintaining fiber (PMF) coil with QAD winding pattern to detect the angular rate Ω. The signal processing circuit incorporates pre-amplifier, AD converter, high-speed field programmable gate array (FPGA) and DA converter. Moreover, the gyroscope sensitivity is maximized by applying a square wave modulation in the IOC at the frequency of 1/2τ, where τ is the propagation time of light in the fiber coil. The gyroscope output data are acquired by Data Acquisition (DAQ) card and then sent into a computer to be stored and post-processed.

**Figure 2:**
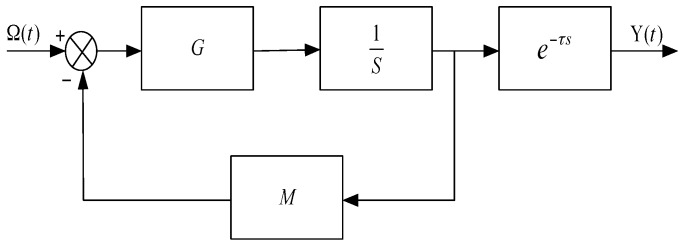
Simplified model of digital closed-loop IFOG.

Based on the control theory and Figure 1, the response of closed-loop IFOG is linear to the angular rate Ω and the dynamic model can be simplified, as shown in Figure 2. *G* is the gain of the gyroscope control system, which is mainly determined by Sagnac coefficient, photo-detector, pre-amplifier, and AD converter. $\frac{1}{S}$ is the integral controller in FPGA. *M* is the feedback coefficient, which is influenced by DA converter and half-wave voltage of IOC, and 1/*M* is the closed-loop scale factor of IFOG. In addition, $e^{-\tau s}$ denotes the delay unit of the control system.

The transfer function of Figure 2 is given as follows.

(1) $$F(s)=\frac{Y(s)}{\Omega (s)}=\frac{K}{Ts+1}e^{-\tau s}$$

Here, K=1/M and T=1/GM. The frequency-amplitude characteristic as well as frequency-phase characteristic of Equation (1) is illustrated as

(2) $$\left\{ \begin{array}{l} \left|F(j\omega )\right|=K\frac{1}{\sqrt{{\left(\omega T\right)}^{2}+1}}\\ \theta [F(j\omega )]=-\arctan(\omega T) \end{array}\right.$$

**Figure 3:**
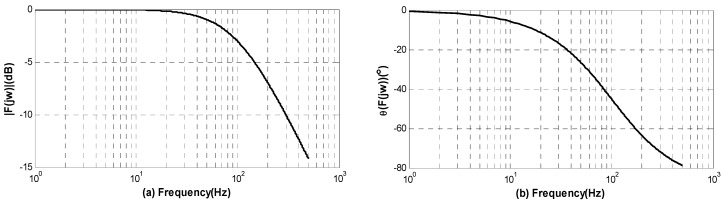
Normalized Bode diagram (**a**) Frequency-amplitude characteristic curve; and (**b**) Frequency-phase characteristic curve.

For a high precision IFOG fabricated in our laboratory, the closed-loop scale factor K is 816 LSB/(°/h) and the time constant T is measured as 1.6 ms. In this case, the normalized f−20lg|F(jω)| curve and f−θ[F(jω)] curve (i.e., Bode diagram) are shown in Figure 3. It is clearly seen from the frequency characteristic curve that the output signal of gyroscope will have a half-attenuation (i.e., −3 dB in Figure 3) at the frequency of 99.47 Hz. Therefore, the vibration-induced rate error of IFOG with high frequency will be dramatically attenuated after passing Equation (1).

## 3. Establishment of Vibration Model

**Figure 4:**
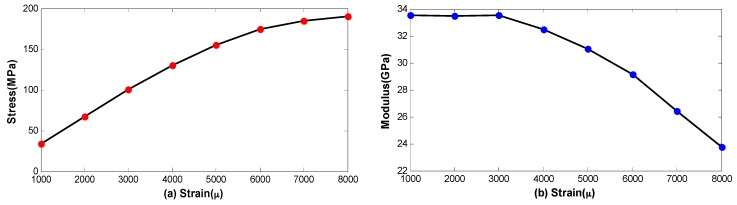
Measurement results of the fiber mechanical properties (**a**) The tensile stress versus the tensile strain; and (**b**) the elastic modulus versus the tensile strain.

In general, the fiber coil has a tensile strain induced by tensile stress, and the strain distribution is asymmetrical with respect to the midpoint of the fiber coil due to imperfect winding techniques. According to the theory of material mechanics, the elastic modulus of the material is not a constant if the fiber strain exceeds a certain range. In this case, different fiber strain has different fiber elastic modulus after winding and we will use the measurement results from [15], as illustrated in Figure 4. Note that the unit of the strain μ denotes the variation of 0.0001% and the curves are fitted by six-order polynomial to guarantee the computational precision. Figure 4 reveals that the relationship between fiber stress and fiber strain is nonlinear if the stain is more than 3000 μ approximately, and the corresponding elastic modulus becomes variable with respect to the fiber strain.

**Figure 5:**
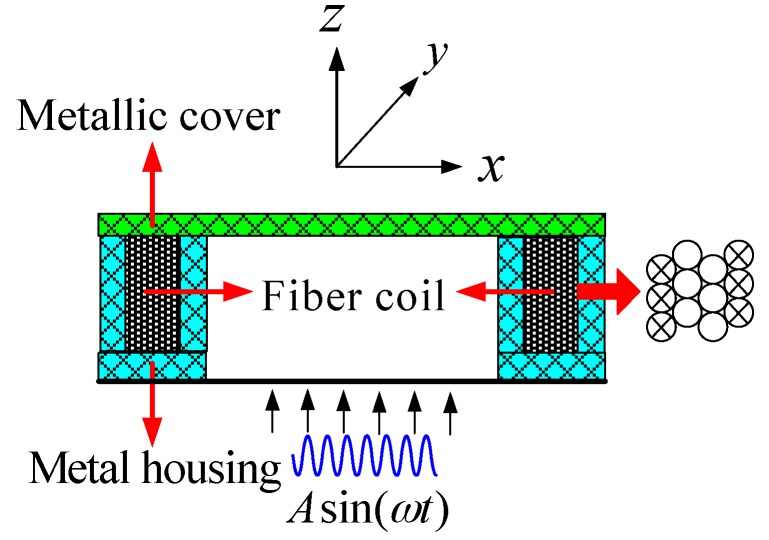
Diagram of the mechanical vibration applied to the fiber coil. The vibration direction is along the *z*-axis and perpendicular to the fiber coil.

Specifically, the fiber coil without spool is solidified by UV-curing adhesive and fixed in the metal housing by metallic cover, as present in Figure 5. The movement of the QAD coil under vibration is along z-axis in a global coordinate system. We assume that the acceleration applied to the coil is described as Asin(ωt), where A is vibration amplitude and ω is angular vibration frequency. Then the velocity and displacement of the coil motion are given as −Acos(ωt)/ω and $-A\sin(\omega t)/\omega^{2}$ by integrating Asin(ωt) one time and two times, respectively; that is

(3) $$\begin{array}{l} \frac{d^{2}z}{dt^{2}}=A\sin(\omega t)\\ \frac{dz}{dt}=-A\cos(\omega t)/\omega \\ z=-A\sin(\omega t)/\omega^{2} \end{array}$$

**Figure 6:**
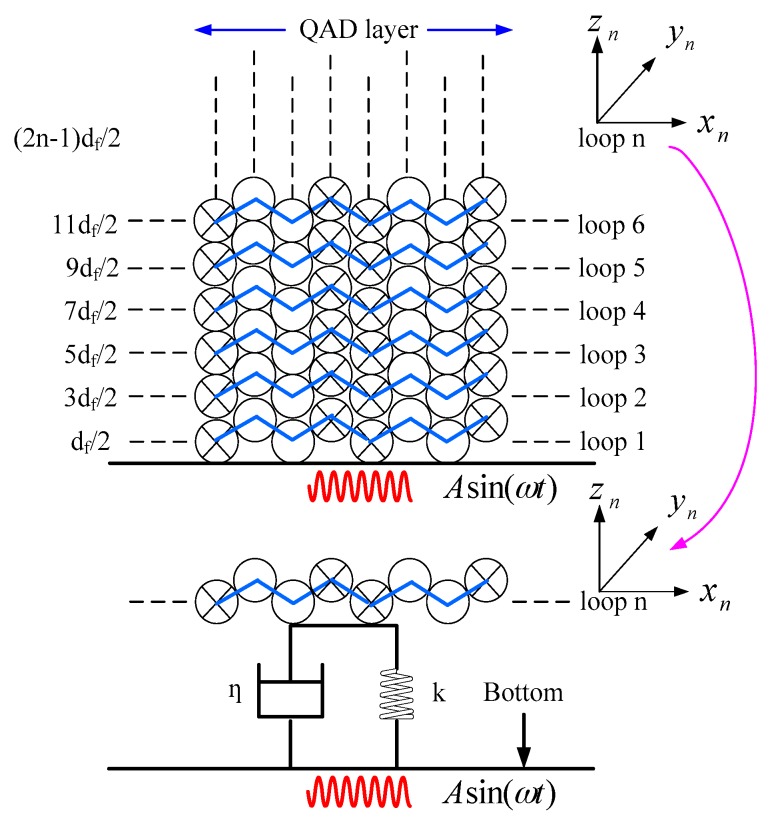
Dynamic model for each fiber loop under vibration.

For a QAD coil with layers number *M* and loops number *N*, the index of each layer and each loop could be denoted by *m* (*m* = 1, 2 … *M*) and *n* (*n* = 1, 2 … *N*). The diameter of the fiber is denoted by $d_{f}$. The dynamic model to illustrate the movement of each loop caused by vibration is shown in Figure 6. To facilitate the analysis, a local coordinate system is established in each loop and the displacement along vibration direction is denoted by $z_{n}$. In this model, each fiber loop is regarded as a mass-spring-damper system with stiffness coefficient *k* and damping coefficient *η*. Here, *η* is a constant for the fiber, while *k* is different in terms of the distance to the coil bottom. The relationship between fiber stress σ and fiber strain ε is

(4) σ=Eε

That is

(5) $$\frac{F}{\Delta S}=E\frac{\Delta L}{L}$$

Then we have

(6) $$F=\frac{E\Delta S}{L}\Delta L=k\Delta L$$

We assume that fibers in Figure 6 are modeled as close-packed with adhesive filling the interstitial volume. In this case, the coil area is calculated by $\Delta S=\pi (R_{2}^{2}-R_{1}^{2})$. Hence, the stiffness coefficient *k* in loop *n* is given as

(7) $$k=\frac{E\pi (R_{2}^{2}-R_{1}^{2})}{\frac{2n-1}{2}d_{f}}$$

where *R_1_* and *R_2_* are inner radius and outer radius of the fiber coil, respectively, and *E* is elastic modulus of the fiber.

Considering that the relative velocity and relative displacement between fiber loop *n* and the coil bottom are described as $\frac{dz_{n}}{dt}-[-\frac{1}{\omega }A\cos(\omega t)]$ and $z_{n}-[-\frac{1}{\omega^{2}}A\sin(\omega t)]$, the motion equations of fiber loop *n* is given in Equation (8). Here, $m_{coil}$ is the coil mass and $\frac{m_{coil}}{N}$ is the mass of each loop.

(8) $$\frac{m_{coil}}{N}\frac{d^{2}z_{n}}{dt^{2}}+\eta [\frac{dz_{n}}{dt}+\frac{A\cos(\omega t)}{\omega }]+\frac{E\pi (R_{2}^{2}-R_{1}^{2})}{\frac{2n-1}{2}d_{f}}[z_{n}+\frac{A\sin(\omega t)}{\omega^{2}}]=0$$

Equation (8) could be rewritten as Equation (9) after rearrangement and being divided by $\frac{m_{coil}}{N}$.

(9) $$-\frac{N\eta }{m_{coil}\omega }A\cos(\omega t)-\frac{\omega_{n}^{2}}{\omega^{2}}A\sin(\omega t)=\frac{d^{2}z_{n}}{dt^{2}}+2\xi \omega_{n}\frac{dz_{n}}{dt}+\omega_{n}^{2}z_{n}$$

In the above equation, $\omega_{n}=\sqrt{\frac{N}{m_{coil}}\frac{E\pi (R_{2}^{2}-R_{1}^{2})}{\frac{2n-1}{2}d_{f}}}$ is the natural resonant frequency, which is usually more than one magnitude larger than ω; and $\xi =\frac{\eta }{2\sqrt{\frac{m_{coil}}{N}\frac{E\pi (R_{2}^{2}-R_{1}^{2})}{\frac{2n-1}{2}d_{f}}}}$ is the damping ratio. It is clearly seen that $\omega_{n}$ and ξ are both determined by loop number *n*. The solution to Equation (9) is given in Equation (10).

(10) $$z_{n}(t)=-\frac{A\sqrt{{m_{coil}}^{2}\omega_{n}^{4}+N^{2}\eta^{2}\omega^{2}}}{m_{coil}\omega^{2}}\frac{\sin(\omega t+\alpha +\beta )}{\sqrt{{\left(\omega_{n}^{2}-\omega^{2}\right)}^{2}+4\xi^{2}\omega_{n}^{2}\omega^{2}}}+C_{1}e^{-\xi \omega_{n}t}\sin(\omega_{n}\sqrt{1-\xi^{2}}t+\gamma )$$

where $\alpha =\tan^{-1}(\frac{N\eta \omega }{m_{coil}\omega_{n}^{2}})$ and $\beta =-\tan^{-1}(\frac{2\xi \omega_{n}\omega }{\omega_{n}^{2}-\omega^{2}})$. Since the second term of Equation (10) will attenuate rapidly after a short period time, the steady state solution of Equation (9) will be the first term of Equation (10). Therefore, the force acting upon coil loop *n* under vibration is described as Equation (11), which is different in different *n* and ω.

(11) $$f_{n}^{vib}=\frac{m_{coil}}{N}\frac{d^{2}z_{n}(t)}{dt^{2}}=\frac{\sqrt{{m_{coil}}^{2}\omega_{n}^{4}+N^{2}\eta^{2}\omega^{2}}/N}{\sqrt{{\left(\omega_{n}^{2}-\omega^{2}\right)}^{2}+4\xi^{2}\omega_{n}^{2}\omega^{2}}}A\sin(\omega t+\alpha +\beta )=F_{n}\sin(\omega t+\alpha +\beta )\;$$

In order to calculate the nonreciprocity phase shift induced by vibration, the whole fiber coil is divided into *M* × *N* segments, and the length of each segment $s_{i}$ based on the cross section of the coil present in Figure 6 is given in Equation (12). In this equation, m is the index of each QAD layer, i is the index of each segment.

(12) $$s_{i}=2\pi [R_{1}+\frac{\sqrt{3}d_{f}}{2}(m-1)](i=1,2,\cdots ,M\times N)$$

According to the material mechanics, the stress employed in each segment should be the force divided by coil area, as shown in Equation (13). The relationship between the index of each loop n and the index of each segment i are also given in Equation (13).

(13) $$\begin{array}{l} P_{i}^{vib}(t)=\frac{F_{n}\sin(\omega t+\alpha +\beta )}{\pi ({R_{2}}^{2}-{R_{1}}^{2})}\;(i=1,2,\cdots ,M\times N)\\ \text{Here},\\ \left\{ \begin{array}{l} n=1\;i=1,N+1,\cdots ,(M-1)N+1\\ n=2\;i=2,N+2,\cdots ,(M-1)N+2\\ \vdots \\ n=N\;i=N,2N,\cdots ,MN \end{array}\right. \end{array}$$

Depending on Figure 4b and Equation (13), the vibrational strain induced by mechanical vibration can be written as

(14) $$\left\{ \begin{array}{ll} \varepsilon_{i}^{vib}(t)=0 & t=0\\ \varepsilon_{i}^{vib}(t)=\frac{P_{i}^{vib}(t)\mu }{E[\varepsilon_{i}^{ten}+\varepsilon_{i}^{vib}(t-\Delta t)]} & t\geq \Delta t \end{array}\right.$$

In Equation (14), $E[\varepsilon_{i}^{ten}+\varepsilon_{i}^{vib}(t-\Delta t)]$ is elastic modulus of the fiber versus tensile strain $\varepsilon_{i}^{ten}$ plus vibrational strain $\varepsilon_{i}^{vib}(t-\Delta t)$ in segment i, Δt is discrete step in time domain, and μ is Poisson’s ratio of the fiber. Therefore, the overall strain in vibrational condition will be the summation of tensile strain and vibrational strain, that is

(15) $$\varepsilon_{i}(t)=\varepsilon_{i}^{ten}+\varepsilon_{i}^{vib}(t)$$

The light with wavelength λ will have a phase shift $\varphi_{i}$ after passing through a fiber with length $s_{i}$ and refractive index $n_{eff\_i}$, i.e., $\varphi_{i}=2\pi n_{eff\_i}s_{i}/\lambda$. In vibrational condition, the length and the refractive index of the fiber coil in segment i will change, as described by Equations (16) and (17) [16], and the corresponding phase shift can be described by Equations (18).

(16) $$s_{i}(t)=s_{i}+s_{i}\varepsilon_{i}(t)$$

(17) $$n_{eff\_i}(t)=n_{eff\_i}+\frac{{n_{eff\_i}}^{3}}{2}[P_{11}\mu -P_{12}(1-\mu )]\varepsilon_{i}(t)$$

(18) $$\begin{array}{ll} \varphi_{i}(t) & =\frac{2\pi }{\lambda }n_{eff\_i}(t)s_{i}(t)=\frac{2\pi }{\lambda }n_{eff\_i}s_{i}+k_{i}\varepsilon_{i}(t)+\frac{{n_{eff\_i}}^{3}s_{i}}{2}[P_{11}\mu -P_{12}(1-\mu )]{\varepsilon_{i}}^{2}(t)\\ & \approx \frac{2\pi }{\lambda }n_{eff\_i}s_{i}+k_{i}\varepsilon_{i}(t) \end{array}$$

where $P_{11}$ and $P_{12}$ are photoelastic coefficients of the fiber and $k_{i}=\frac{2\pi }{\lambda }n_{eff\_i}s_{i}+\frac{\pi {n_{eff\_i}}^{3}}{\lambda }s_{i}[P_{11}\mu -P_{12}(1-\mu )]$. Note that the last term of Equation (18) is ignored since it is very small as compared with the former three terms. Substituting Equation (13) into Equations (14) and (15), Equation (18) could be rewritten as Equation (19). Note that we only consider the situation t≥Δt, which means $\varepsilon_{i}^{vib}(t)$ exists

(19) $$\varphi_{i}(t)=\frac{2\pi }{\lambda }n_{eff\_i}s_{i}+k_{i}\{\varepsilon_{i}^{ten}+\frac{\frac{F_{i}\sin(\omega t+\alpha +\beta )}{\pi ({R_{2}}^{2}-{R_{1}}^{2})}\;\mu }{E[\varepsilon_{i}^{ten}+\varepsilon_{i}^{vib}(t)]}\}$$

Depending on Equation (19), the light will have a phase shift $\varphi_{cw}$ and $\varphi_{ccw}$ after passing through the whole optical path in clockwise and counterclockwise directions respectively, as described by Equations (20) and (21). In this case, the equivalent rate error of IFOG induced by mechanical vibration can be calculated by Equation (22) according to Sagnac effect.

(20) $$\varphi_{cw}(t)=\frac{2\pi }{\lambda }n_{eff\_i}L+\sum_{i=1}^{M\times N}k_{i}\{\varepsilon_{i}^{ten}+\frac{F_{i}\sin[\omega (t+\frac{s_{1}+s_{2}+\cdots s_{i}}{c})+\alpha +\beta ]\mu }{\pi ({R_{2}}^{2}-{R_{1}}^{2})E[\varepsilon_{i}^{ten}+\varepsilon_{i}^{vib}(t)]}\}$$

(21) $$\varphi_{ccw}(t)=\frac{2\pi }{\lambda }n_{eff\_i}L+\sum_{i=1}^{M\times N}k_{M\times N+1-i}\{\varepsilon_{M\times N+1-i}^{ten}+\frac{F_{M\times N+1-i}\sin[\omega (t+\frac{s_{M\times N}+s_{M\times N-1}+\cdots +s_{M\times N+1-i}}{c})+\alpha +\beta ]\mu }{\pi ({R_{2}}^{2}-{R_{1}}^{2})E[\varepsilon_{M\times N+1-i}^{ten}+\varepsilon_{M\times N+1-i}^{vib}(t)]}\}$$

**Figure 7:**
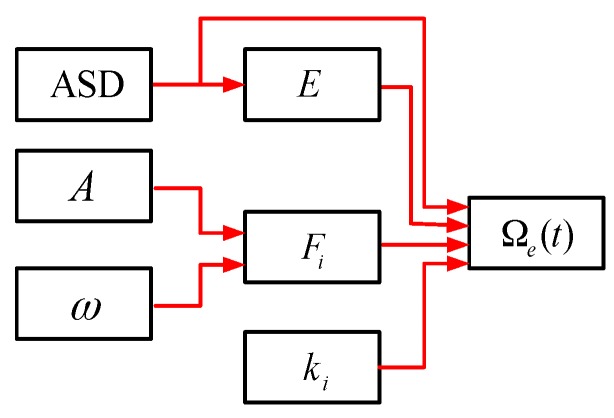
Factors that influence IFOG vibration error.

(22) Ωe(t)=[φcw(t)−φccw(t)]λc2πLD180×3600π(oh)

where c is velocity of the light in vacuum, D is mean diameter of the fiber coil, and $L=\sum_{i=1}^{M\times N}s_{i}$ is the length of the coil. For further analysis and discussion, the definition of asymmetry of strain distribution (ASD) is given in Equation (23). According to the definition, ASD has no unit and can be used as a specification to evaluate the winding quality of the fiber coil. The lower ASD, the better the coil will be. We can see from Equation (11) to Equation (22) that the vibration error $\Omega_{e}(t)$ is determined by the factors that illustrated in Figure 7.

(23) $$\text{ASD}=\sum_{i=1}^{\frac{M\times N}{2}}\left|\varepsilon_{i}-\varepsilon_{M\times N+1-i}\right|$$

Based on Figure 7, we can see that the vibration error $\Omega_{e}(t)$ is determined by internal factors include ASD, E and $k_{i}$, as well as external factors include A and ω. For a given vibration condition, $\Omega_{e}(t)$ can be significantly suppressed if the fiber strain satisfies Equation (24) as follows (i.e., ASD=0). Besides, $\Omega_{e}(t)$ can also be suppressed effectively if the fiber elastic modulus E keeps constant or varies slightly in the whole range of the fiber strain (i.e., less than 3000 μ in Figure 4), as shown in Equation (25). However, it should be pointed out that the parameter in each loop $k_{i}$ is asymmetrical with respect to the coil center since the characterization of QAD pattern. Hence, the residual errors will exit even when the coil fully meet Equation (24), as will be seen from the simulation result in Section 4.

(24) $$\varepsilon_{i}^{ten}=\varepsilon_{M\times N+1-i}^{ten}(i=1,2,\cdots ,\frac{M\times N}{2})$$

(25) $$E[\varepsilon_{i}^{ten}]=\text{constant}(i=1,2,\cdots ,M\times N)$$

## 4. Computation

**Figure 8:**
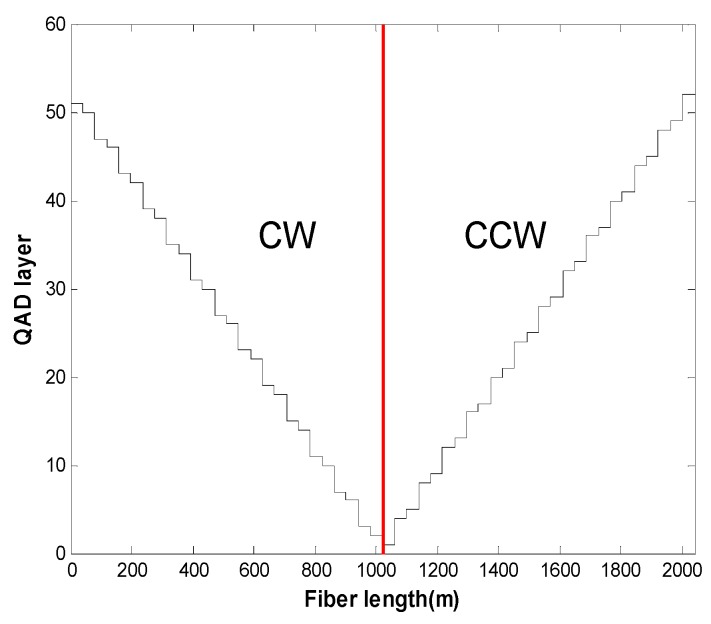
Standard representation of the quadrupolar (QAD) winding pattern.

Geometric parameters and material properties of the fiber coil for theoretical computation are listed in Table 1 and Table 2 [6,16], respectively. Figure 8 shows the standard representation of the QAD fiber coil, where CW and CCW refer to the fiber layer wound in clockwise and counterclockwise directions.

**Figure 9:**
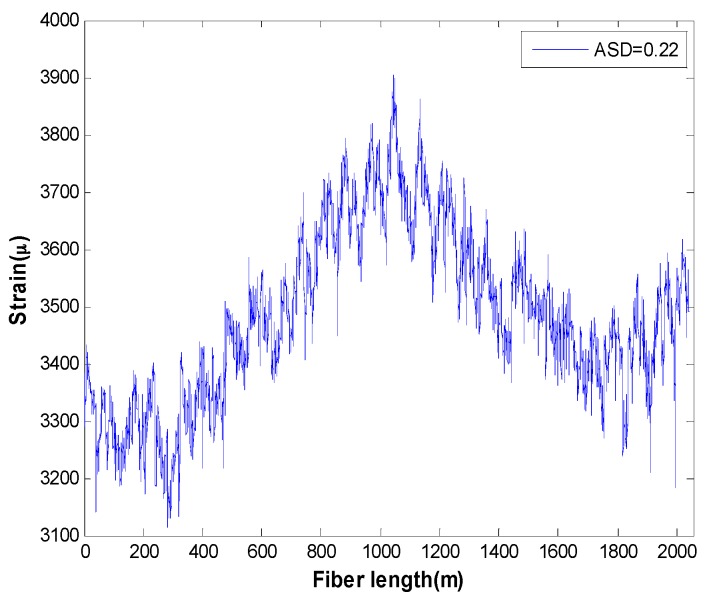
Strain distribution of the QAD fiber coil.

Considering that the computation of the model is based on the measurement data of the fiber strain, a stress analyzer NBX6055PM produced by Neubrex Corporation is utilized to obtain the strain distribution of the fiber coil, as shown in Figure 9. The sampling interval of the stress analyzer along the fiber coil is 5 cm. Figure 9 clearly reveals that the strain distribution is asymmetrical with respect to the midpoint of the fiber coil due to imperfect winding. In this case, the specification ASD is essential to evaluate the asymmetry of the coil, and the calculated result in terms of Equation (23) is 0.22.

As aforementioned, the vibration error $\Omega_{e}(t)$ is mainly dependent on A, ω and ASD. Therefore, the undesired error of IFOG induced by vibration is actually described by the function as follows. Based on Equation (26), the simulations are performed according to different parameters, A, ω and ASD.

(26) $$\Omega_{e}(t)=\Omega_{e}(A,\omega ,\text{ASD},t)$$

**Figure 10:**
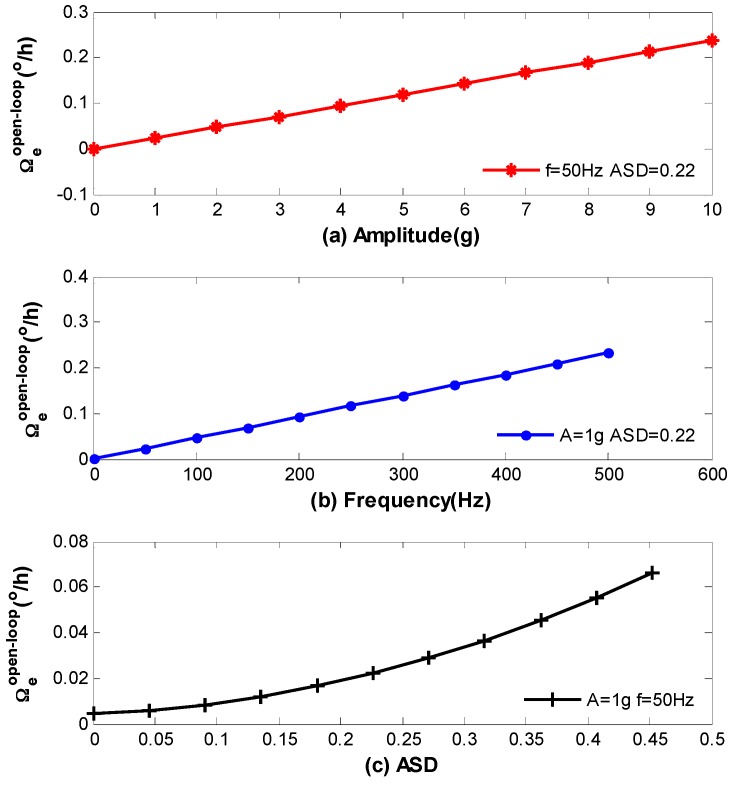
Standard deviation of vibration errors for open-loop IFOG (**a**) Open-loop errors versus amplitude; (**b**) Open-loop errors versus frequency; (**c**) Open-loop errors versus ASD.

Firstly, the open-loop vibration error is calculated through Equations (3)–(22), that is Equation (28) as follows, and the computational results are present in Figure 10. Here, the vibration amplitudes in Figure 10a range from 0 g to 10 g, the vibration frequency is fixed at 50 Hz, and the strain in the coil is shown in Figure 9 with ASD=0.22. The vibration frequencies in Figure 10b range from 0 Hz to 500 Hz, the vibration amplitude is fixed at 1 g, and the strain in the coil is also shown in Figure 9 with ASD=0.22.

To simulate the relationship between $\Omega_{e}(t)$ and different ASD in Figure 10c, we assume the strain in the coil from starting point to midpoint is identical with Figure 9, while the strain from the ending point to midpoint is given in Equation (27).

(27) $$\varepsilon_{M\times N+1-i}=\varepsilon_{i}+\Delta \varepsilon (\text{i}=1,2,\cdots ,\frac{M\times N}{2})$$

where Δε=0μ,20μ,40μ,⋯,200μ, and the corresponding ASD can be obtained in terms of the definition in Equation (23). The vibration amplitude and frequency are fixed at 1 g and 50 Hz respectively.

**Figure 11:**
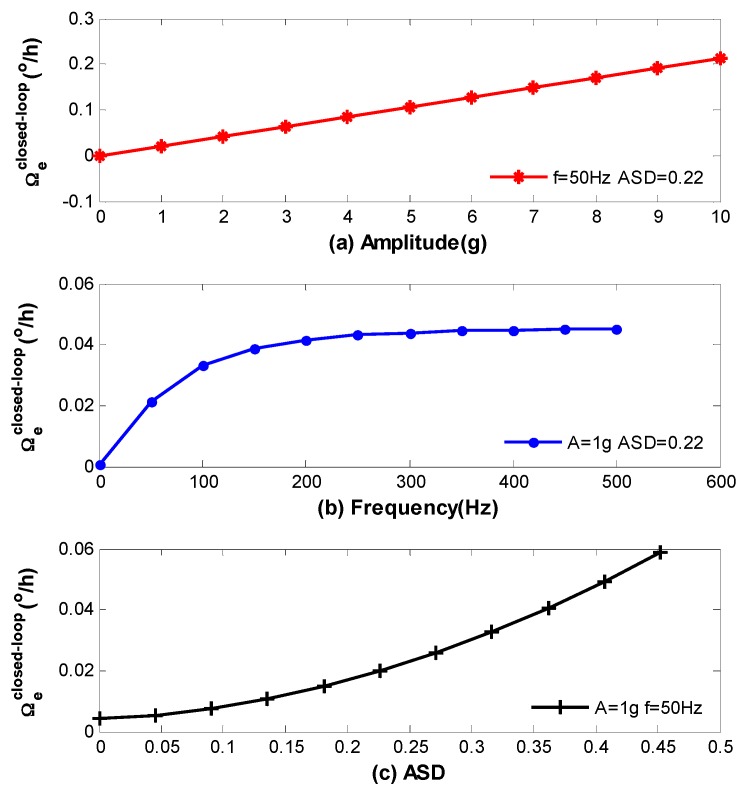
Standard deviation of vibration errors for closed-loop IFOG (**a**) Closed-loop errors versus amplitude; (**b**) Closed -loop errors versus frequency; (**c**) Closed -loop errors versus ASD.

Secondly, the closed-loop vibration error is achieved through Equations (1) and (28) as described by Equation (29) as follows, where $L^{-1}$ denotes Laplace inverse transformation, and the results are present in Figure 11. It should be noted that the computational results of Equations (28) and (29) are present in the form of sinusoidal function, which has the same frequency with vibration. Figure 10 and Figure 11 only show the standard deviation of the errors.

(28) $${\Omega_{e}}^{open-loop}(t)=\Omega_{e}(t)$$

(29) $${\Omega_{e}}^{closed-loop}(t)=\Omega_{e}(t)L^{-1}(\frac{1}{Ts+1}e^{-\tau s})$$

We can see from the above figures that vibration errors in both open-loop and closed-loop IFOG increase with the raise of A, ω and ASD. However, there are still residual errors in Figure 10c and Figure 11c when ASD=0. As has been discussed before, this is because the coefficient $k_{i}$ in each loop is asymmetrical with respect to the coil center due to the characterization of QAD pattern. Moreover, some model’s error may exit which can affect theoretical values in Figure 10 and Figure 11. The reasons are parameters in Table 1 and Table 2 are not fully accurate and the strain distribution shown in Figure 9 could be influenced by temperature. Nevertheless, the results are, to some extent, consistent with the experimental measurements in [11].

**Figure 12:**
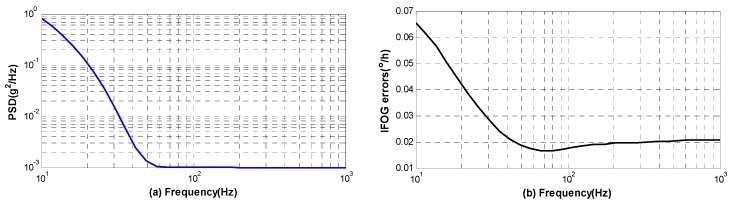
PSD of vibration in F-15B aircraft and the corresponding standard deviation of errors in IFOG (**a**) PSD of vibration; and (**b**) IFOG errors versus PSD.

To provide a quantitative estimation of the performance of IFOG in an environment with high level vibration, the errors of IFOG are computed based on the model under the power spectral density (PSD) that obtained by accelerometers mounted in NASA F-15B aircraft [17]. As has been studied in [17], the lateral axis acceleration was found to be dominant for most of the flight conditions. Hence, we will simulate the vibration in lateral direction when the aircraft takeoff. The PSD between 10 Hz and 1 kHz is plotted in Figure 12 with blue line and the corresponding vibration errors are also plotted with black line. To facilitate the calculation based on the mathematical model, we firstly calculate root mean square (RMS) of vibration amplitude in each frequency according to PSD, and then the random vibration could be transformed into the deterministic signal.

The result indicates that vibration errors are mostly generated by low-frequency vibration between 10 Hz and 60 Hz, and the largest error in IFOG is approximately 0.065°/h. Moreover, the total value of vibration error is about 0.12°/h by integrating the PSD. In this case, it is essential to improve the mechanical design of gyro to isolate the vibration between 10 Hz and 60 Hz. This will be meaningful to achieve a better navigation precision when the aircraft takeoff.

## 5. Conclusions

In this paper, the mathematical model of IFOG under vibration is established based on the strain distribution of the QAD fiber coil. Theoretical analysis indicates that the asymmetrical strain distribution in the fiber coil and the variable fiber elastic modulus in large strain situation are two dominant reasons that give rise to rate errors of IFOG in vibrational environment. Moreover, the errors will increase with the raise of vibrational amplitude, frequency and ASD. Finally, an estimation of vibration-induced IFOG errors in NASA aircraft is done based on the proposed model.

In summary, our work is meaningful to achieve a more robust (against mechanical vibration) sensor performance for high precision IFOG working in harsh environments.

## Figures and Tables

**Table 1:** Geometric parameters.

Parameters	Values
Fiber coil length *L*	2058 m
Layers number *M*	52
Loops number *N*	87
Inner radius *R_1_*	68 mm
Outer radius *R_2_*	76.8 mm
Fiber diameter *d_f_*	169 μm

**Table 2:** Material properties.

Parameters	Values
Refractive index *n_eff_i_* Damping coefficient *η*	1.45
	0.01
Poisson‘s ratio *μ*	0.17
Photoelastic coefficients *P_11_* and *P_12_*	0.121 and 0.270
Fiber coil mass *m_coil_*	69.94 g
Light wavelength *λ*	1550 nm

## References

[1] Pavlath G.A. Fiber optic gyros past, present, and future. Proceedings of the SPIE OFS2012 22nd International Conference on Optical Fiber Sensor.

[2] Sanders G.A., Szafraniec B., Liu R.Y., Bielas M.S., Strandjord L.K. Fiber-optic gyro development for a broad range of applications. Proceedings of the SPIE European Symposium on Optics for Environmental and Public Safety.

[3] Sanders G.A., Szafraniec B., Liu R.Y., Laskoskie C.L., Strandjord L.K., Weed G. Fiber optic gyros for space, marine, and aviation applications. Proceedings of the SPIE‘s 1996 International Symposium on Optical Science, Engineering, and Instrumentation.

[4] Ciminelli C., Dell’Olio F., Campanella C.E., Armenise M.N. (2010). Photonic technologies for angular velocity sensing. Adv. Opt. Photonics.

[5] Lefèvre H.C. (2013). The fiber-optic gyroscope: Challenges to become the ultimate rotation-sensing technology. Opt. Fiber Technol..

[6] Zhang Y., Gao Z., Wang G., Gao W. (2014). Modeling of thermal-induced rate error for FOG with temperature ranging from −40 °C to 60 °C. IEEE Photonics Technol. Lett..

[7] Kim H.K., Digonnet M.J.F., Kino G.S. (2006). Air-core photonic-bandgap fiber-optic gyroscope. J. Lightw. Technol..

[8] Blin S., Kim H.K., Digonnet M.J., Kino G.S. (2007). Reduced thermal sensitivity of a fiber-optic gyroscope using an air-core photonic-bandgap fiber. J. Lightw. Technol..

[9] Webber M., Willig R., Raczkowski H., Dineen A. (2012). Modeling of rate error in interferometric fiber-optic gyroscopes due to stress induced by moisture diffusion. J. Lightw. Technol..

[10] Wu L., Cheng J.H., Sun F. Research on anti-vibration scheme of FOG SINS. Proceedings of the International Conference Computer Engineering and Technology (ICCET).

[11] Song N.F., Zhang C.X., Du X.Z. Analysis of vibration error in fiber optic gyroscope. Proceedings of the SPIE Photonics Asia 2002.

[12] Moslehi B., Yahalom R., Oblea L., Faridian F., Black R.J., Ha J.C., Berarducci M. Low-cost and compact fiber-optic gyroscope with long-term stability. Proceedings of the Aerospace Conference.

[13] Li Z., Meng Z., Wang L., Liu T., Yao S.X. (2015). Tomographic inspection of fiber coils using optical coherence tomography. IEEE Photoics Technol. Lett..

[14] Kurbatov A.M., Kurbatov R.A. (2013). The vibration error of the fiber-optic gyroscope rotation rate and methods of its suppression. J. Commun. Technol. Electron..

[15] Hudson S.D., Zhurov V., Grbić V., Grbić M., Hutter J.L. (2013). Measurement of the elastic modulus of spider mite silk fibers using atomic force microscopy. J. Appl. Phys..

[16] Zhang Y.G., Gao Z.X. (2012). Fiber optic gyroscope vibration error due to fiber tail length asymmetry based on elastic-optic effect. Opt. Eng..

[17] Corda S., Franz R.J., Blanton J.N., Vachon M.J., DeBoer J.B. (2002). In-Flight Vibration Environment of the NASA F-15B Flight Test Fixture.

